# Cysteine and glycine-rich protein 2 (*CSRP2*) transcript levels correlate with leukemia relapse and leukemia-free survival in adults with B-cell acute lymphoblastic leukemia and normal cytogenetics

**DOI:** 10.18632/oncotarget.16416

**Published:** 2017-03-21

**Authors:** Shu-Juan Wang, Ping-Zhang Wang, Robert Peter Gale, Ya-Zhen Qin, Yan-Rong Liu, Yue-Yun Lai, Hao Jiang, Qian Jiang, Xiao-Hui Zhang, Bin Jiang, Lan-Ping Xu, Xiao-Jun Huang, Kai-Yan Liu, Guo-Rui Ruan

**Affiliations:** ^1^ Peking University People's Hospital and Institute of Hematology, Beijing Key Laboratory of Hematopoietic Stem Cell Transplantation, Beijing, China; ^2^ Department of Immunology, School of Basic Medical Sciences, Peking University Health Science Center, Key Laboratory of Medical Immunology, Ministry of Health, China, Peking University Center for Human Disease Genomics, Beijing, China; ^3^ Hematology Research Center, Division of Experimental Medicine, Department of Medicine, Imperial College London, London, UK; ^4^ Peking-Tsinghua Center for Life Sciences, Beijing, China

**Keywords:** acute lymphoblastic leukemia, CSRP2, prognostic factor, relapse, drug resistance

## Abstract

Relapse is the major cause of treatment-failure in adults with B-cell acute lymphoblastic leukemia (ALL) achieving complete remission after induction chemotherapy. Greater precision identifying persons likely to relapse is important. We did bio-informatics analyses of transcriptomic data to identify mRNA transcripts aberrantly-expressed in B-cell ALL. We selected 9 candidate genes for validation 7 of which proved significantly-associated with B-cell ALL. We next focused on function and clinical correlations of the cysteine and glycine-rich protein 2 (*CSRP2*). Quantitative real-time polymerase chain reaction (RT-qPCR) was used to examine gene transcript levels in bone marrow samples from 236 adults with B-cell ALL compared with samples from normals. *CSRP2* was over-expressed in 228 out of 236 adults (97%) with newly-diagnosed B-cell ALL. A prognostic value was assessed in 168 subjects. In subjects with normal cytogenetics those with high *CSRP2* transcript levels had a higher 5-year cumulative incidence of relapse (CIR) and worse relapse-free survival (RFS) compared with subjects with low transcript levels (56% [95% confidence interval, 53, 59%] vs. 19% [18, 20%]; *P* = 0.011 and 41% [17, 65%] vs. 80% [66–95%]; *P* = 0.007). In multivariate analyses a high *CSRP2* transcript level was independently-associated with CIR (HR = 5.32 [1.64–17.28]; *P* = 0.005) and RFS (HR = 5.56 [1.87, 16.53]; *P* = 0.002). Functional analyses indicated *CSRP2* promoted cell proliferation, cell-cycle progression, *in vitro* colony formation and cell migration ability. Abnormal *CSRP2* expression was associated with resistance to chemotherapy; sensitivity was restored by down-regulating *CSRP2* expression.

## INTRODUCTION

B-cell acute lymphoblastic leukemia (ALL) is characterized by clonal expansion of developmentally-arrested B-cell precursors [1]. Although survival of adults with B-cell ALL has improved relapse is an important problem. Prognostic models for relapse include age, WBC levels at diagnosis, immune phenotype, cytogenetics, mutational landscape, response to induction therapy and measureable residual disease (MRD) after completing therapy [2]. Adverse cytogenetic and mutations include hypo-diploidy (< 44 chromosomes), *MLL*/11q23 translocations, complex cytogenetics (≥ 5 abnormalities) and t (9; 22) and/or *BCR-ABL1* [2]. However, about one-half of adults with B-cell ALL have none of the adverse prognostic variables at diagnosis making predicting relapse difficult, especially so in those with normal cytogenetics [3, 4]. Identifying a new prognostic variable in these persons is important [5].

Analyzing differential expression of mRNAs is a new approach to predicting outcomes of persons with B-cell ALL. For example, in adults with B-cell ALL increased CTGF (connective tissue growth factor) and *LEF1* (lymphoid enhancer binding factor-1) expression are associated with worse RFS [6, 7] whereas increased *BAALC* (brain and acute leukemia, cytoplasmic) expression is associated with an unfavorable response to chemotherapy and worse survival [8].

A bioinformatics-based evaluation of candidate mRNAs improves efficiency compared with random sampling [9]. We used publicly available genome-wide mRNA expression data from patients with B-cell ALL to identify differentially expressed transcripts compared with normals. We identified 9 candidate genes 7 of which we validated and focused our attention on *CSRP2* (cysteine and glycine-rich protein 2). *CSRP2* is a member of *CSRP* family encoding a group of short LIM domain proteins (21 kDa) which are critical regulators of development and differentiation [10]. The three CSRPs (CSRP1-3) are preferentially expressed in muscle cells localizing to the nucleus and cytoplasm [11]. In the nucleus, they facilitate smooth muscle differentiation via interactions with transcription factors [12]. In the cytoplasm they decorate filamentous actin structures and participate in cytoskeletal remodeling [13]. *CSRP2* maps to 12q21 which is reported abnormal in haematological neoplasms including T-cell ALL and lymphoma [14–16]. Increased *CSRP2* transcript levels are associated with dedifferentiation in hepatocellular carcinoma [17]. In microarray-based analyses high-expression of *CSRP2* is associated with basal-like breast cancer [18, 19]. However, there were no reports regarding the role of *CSRP2* in hematological neoplasms. Here, we studied levels of *CSRP2* transcripts for an association with relapse probability in adults with B-cell ALL. We show increased *CSRP2* transcript levels are independently-associated with higher cumulative incidence of relapse (CIR) and worse relapse-free survival (RFS) in adults with B-cell ALL and normal cytogenetics.

## RESULTS

### Validation of new biomarkers for B-cell ALL based on genome-wide mRNA analyses

We studied differentially-expressed genes in normal and B-cell ALL using data from the ImmuSort database (http://immusort.bjmu.edu.cn; Table 1). We focused on the top 20 differentially expressed genes based on the delta values > 45 and average rank scores (ARSs) > 80 in B-cell ALL samples. To increase reliability of our analyses we updated these data with relevant data from the Gene Expression Omnibus (GEO) [9]. The final dataset was based on 400 B-cell samples (GEO samples/GSMs, arrays or measurements) from normals and 690 samples from persons with B-cell ALL and confirmed our target gene selection.

**Table 1:** Gene expression levels of the selected top 20 genes with differential expression

	Gene ID	B-cell ALL	B-cell	Delta	B-cell ALL^a^	B-cell^a^	Delta^a^	B-cell CLL
***DNTT***	1791	97.3	45.25	52.05	99.00	47.28	51.72	42.39
***C5orf62^b^***	85027	97.13	47.78	49.35	97.11	50.27	46.84	52.62
***GNA15^b^***	2769	94.21	44.21	50	95.02	45.13	49.89	47.05
***VPREB1***	7441	93.07	30.73	62.34	89.57	33.17	56.40	25.72
***CSRP2b***	1466	91.75	43.77	47.98	92.55	43.16	49.39	38.69
***ERG***	2078	90.97	36.54	54.43	92.32	34.78	57.54	35.76
***FLT3***	2322	90.64	37.59	53.05	91.01	38.50	52.51	56.06
***IGFBP7***	3490	90.24	35.91	54.33	88.47	33.91	54.56	44.68
***HBEGF^b^***	1839	89.86	39.48	50.38	90.75	41.42	49.33	42.88
***SPRY2***	10253	89.27	38.67	50.6	89.73	33.85	55.88	60.34
***RASD1^b^***	51655	89.25	41.85	47.4	92.93	40.56	52.37	52.62
***CPNE2^b^***	221184	89.24	37.13	52.11	89.45	37.14	52.31	40.25
***ZNF423***	23090	87.66	23.05	64.61	86.55	22.73	63.82	20.50
***FRMD4B^b^***	23150	87.47	28.57	58.9	89.47	27.75	61.72	36.59
***DBN1***	1627	85.85	37.34	48.51	87.18	38.32	48.86	48.35
***CLEC11A***	6320	85.67	36.54	49.13	87.63	41.00	46.63	29.93
***C19orf77^b^***	284422	84.8	30.51	54.29	84.85	34.01	50.84	28.16
***SLC22A16***	85413	84.63	28.3	56.33	85.99	27.33	58.66	30.61
***CTGF***	1490	83.18	31.71	51.47	83.32	36.01	47.31	14.16
***COL5A1^b^***	1289	81.38	31.69	49.69	85.45	36.79	48.66	29.17

^a^Updated B-cell ALL (690 GSMs) and normal B-cell (400 GSMs) samples. The rest represent samples from B-cell ALL (314 GSMs), B-cell chronic lymphoid leukemia (B-cell CLL; 767 GSMs) and B-cells without B-cell ALL and B-cell CLL samples (B-cell; 464 GSMs include 400 samples from normals and 64 samples from other diseases).

^b^9 selected genes without functional reports relevant to B-cell ALL. Values indicate average rank scores (ARSs) of genes in their respective samples.

**Figure 1:**
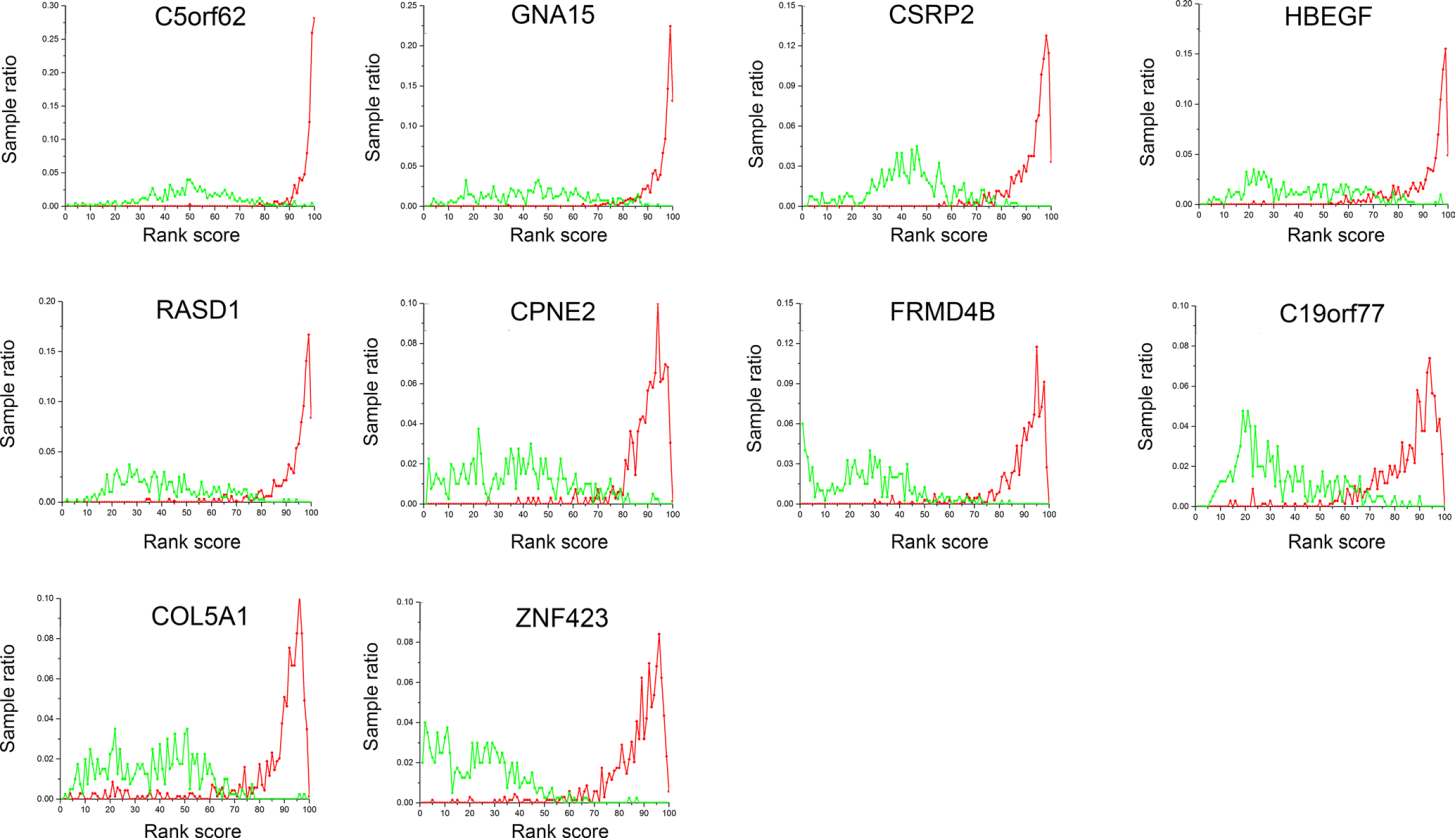
Gene expression profiles of the 9 selected genes Rank-based gene expression (RBE) curves discussed in the ImmuSort database indicate sample distribution in terms of gene expression across various individuals and experimental conditions. The x-axis represents the percentile rank scores from 1 to 100 with increasing expression intensity. The y-axis represents the sample proportion at an indicated rank score. The right and left peaks indicate high and low expression, respectively. Green lines are B cells from normals and red lines, B-cells from B-cell ALL. *ZNF423* is illustrated as a positive control.

Differentially-expressed genes meeting our threshold included *CTGF* (connective tissue growth factor), *ZNF423* (zinc finger protein 423), *VPREB1* (pre-B lymphocyte 1), *SLC22A16* (solute carrier family 22 member 16), *ERG* (ETS transcription factor), *IGFBP7* (insulin like growth factor binding protein 7), *FLT3* (fms related tyrosine kinase-3), *DNTT* (DNA nucleotidyl exotransferase), *SPRY2* (sprouty RTK signaling antagonist 2), *CLEC11A* (C-type lectin domain family 11 member A) and *DBN1* (drebrin-1). Expression of several of these genes such as *CTGF* and *DNTT* (*TdT*) are reported to correlate with therapy-outcomes of persons with B-cell ALL [6, 20–29]. Consequently, we focused on 9 previously-unstudied genes including *C5orf62* (*SMIM3*; small integral membrane protein 3), *GNA15* (G-protein subunit alpha 15), *CSRP2*, *HBEGF* (heparin binding *EGF* like growth factor), *RASD1* (RAS-related dexamethasone induced-1), *CPNE2* (copine-2), *FRMD4B* (*FERM* domain containing 4B), *C19orf77* (*SMIM24*; small integral membrane protein 24) and *COL5A1* (collagen type-V alpha-1 chain; Table 1 and Figure 1).

**Figure 2:**
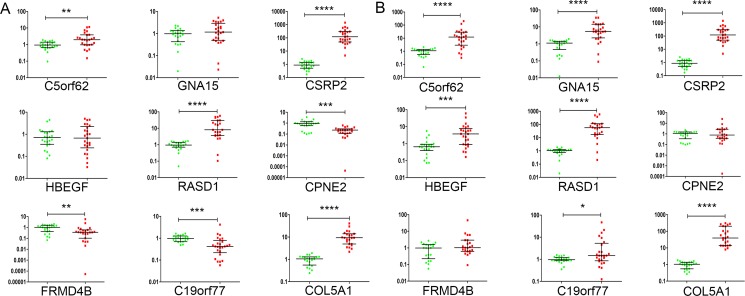
Validation of the 9 selected genes by RT-qPCR (**A**) Transcript levels with *ABL1* as the internal control. (**B**) Transcript levels with *GAPDH* as the internal control. The y-axis represents relative transcript level of genes. Green dots are normal bone marrow samples and red dots, adults with B-cell ALL. Bars represent median and quartiles value. *P < 0.05; **P < 0.01; ***P < 0.001; ****P < 0.0001.

Next we used RT-qPCR to verify differential mRNA levels of these genes in bone marrow cells from 26 adults with newly-diagnosed B-cell ALL compared with cells from 23 normals (Figure 2A). mRNA levels of *CSRP2*, *COL5A1*, *RASD1* and *C5orf62* were significantly increased whereas *HBEGF*, *GNA15*, *FRMD4B*, *C19orf77* and *CPNE2* were not.

Our re-analysis of cases of B-cell ALL in ImmuSort revealed most samples were from children with B-cell ALL. Consequently, we re-searched the GEO database and found the GSE34861 dataset was from adults with B-cell ALL (191 GSMs) [30]. However, this dataset was not derived from the Affymetrix Human Genome U133 Plus 2.0 Array platform (GEO platform/GPL570) but from the NimbleGen Human Expression Array (GPL15088) despite the fact both platforms include genome-wide transcriptome arrays. We next rank-normalized the dataset to derive ARS values for these genes as described [9]. We found all genes shown in Table 1 except *C19orf77* not included in the GPL15088 platform were also up-regulated. *FRMD4B* had the lowest ARS value (61.76) and the remaining genes had ARS values > 75.

Because our RT-qPCR validation studies used *ABL1* as an internal control we compared *ABL1* expression in children and adults with B-cell ALL. *ABL1* mRNA levels were dramatically higher in adults with B-cell ALL with ARS ≥ 93 compared with children B-cell ALL with ARS ≥ 86 (Supplementary Figure 1). Consequently, we switched to a *GAPDH* (glyceraldehyde-3-phosphate dehydrogenase) internal control and re-studied 9 genes 7 of which were up-regulated (Figure 2B). There were insufficient data to critically-analyze *FRMD4B* and *CPNE2* which had Q3 values which skewed higher than controls (Figure 2B). *CSRP2* was the most differentially expressed gene in our validation studies (Figure 2).

### *CSRP2* transcript levels in B-cell ALL

**Figure 3:**
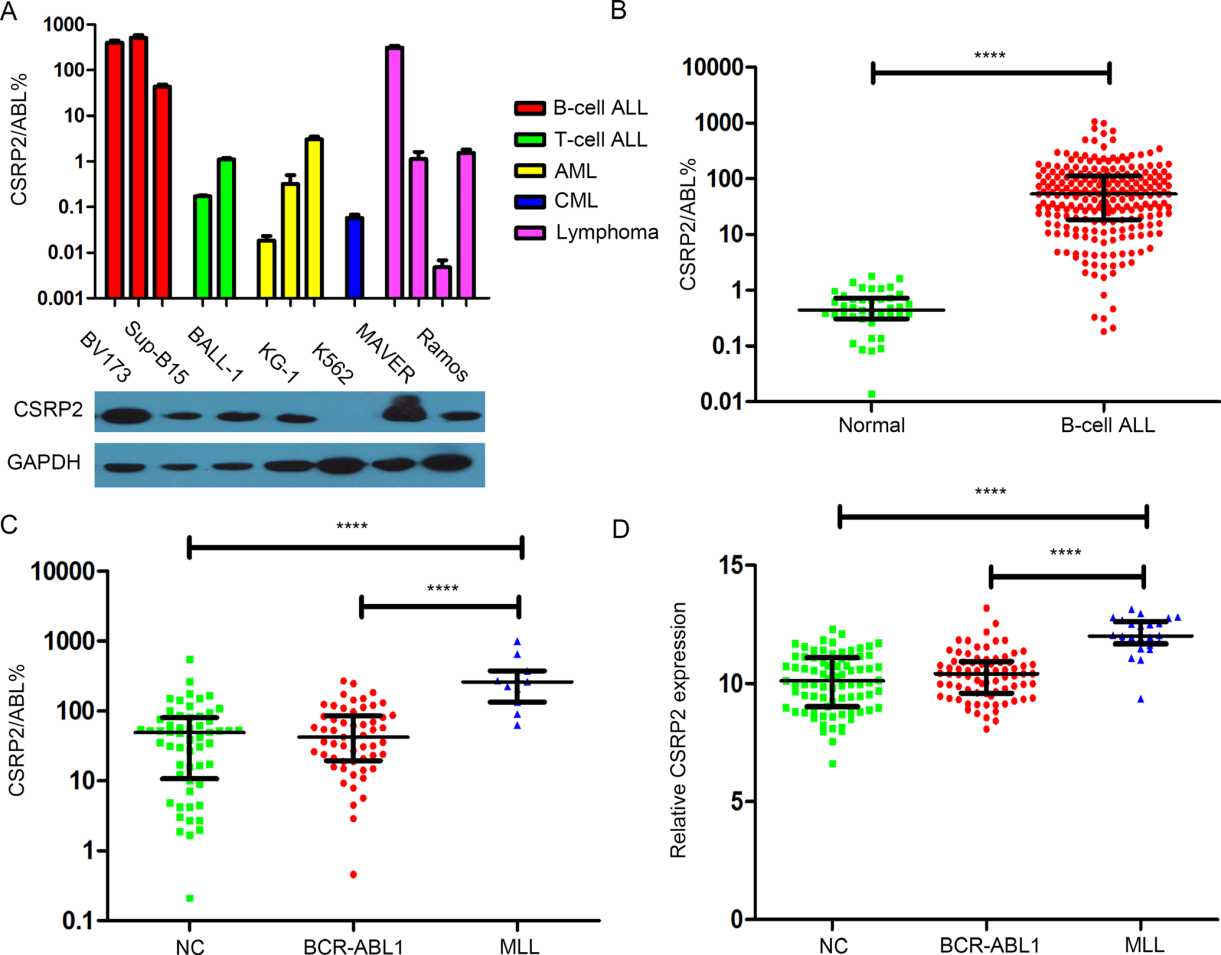
*CSRP2* transcript level *CSRP2* expression in (**A**) Leukemia and lymphoma cell lines (up: RT-qPCR; down: western blot); (**B**) Normal bone marrow and adults with B-cell ALL; (**C**) Different karyotypes of adults with B-cell ALL; (**D**) Different karyotypes of adults with B-cell ALL from the data set of GSE34861. Bars represent median and quartiles value. NC, normal cytogenetics. ****P < 0.0001.

*CSRP2* transcript levels were significantly higher in B-cell ALL cell lines (BV173, Sup-B15 and BALL-1) and a mantle cell lymphoma cell line (MAVER) compared with T-cell ALL cell lines (6T-CEM, MOLT4), a chronic myeloid leukemia cell line (K562), acute myelogenous leukemia (AML) cell lines (KG-1, NB4, HL60) or other lymphoma cell lines (U937, Raji, Ramos; Figure 3A and Supplementary Table 1).

Next we studied bone marrow samples from subjects with B-cell ALL at diagnosis. *CSRP2* transcript levels were significantly higher (median 53%; range 0 – 1066%) compared with levels in 43 normals (0.44%; 0 – 1.78%; *P* < 0.0001; Figure 3B). Receiver-operator characteristic (ROC) curve analyses identified an area under the curve (AUC) for *CSRP2* transcript levels of 0.980 (95% confidence interval [CI], 0.964, 0.996; *P* < 0.0001) with a maximum *CSRP2* transcript level Youden index of 1.83%. Using this cut-off value the rate of *CSRP2* over-expression in newly-diagnosed adult B-cell ALL was 97%. Subjects with *MLL* translocation (*N* = 11; median 262%; range 63–995%) had the highest *CSRP2* transcript levels. Subjects with a normal cytogenetics (*N* = 56; 49% [0.21, 548%]) or *BCR-ABL1* (*N* = 56; 42% [0.46, 269%]) had lower *CSRP2* transcript levels (Figure 3C). These results agree with the data set of GSE34861 [30] which included samples from 191 adults with B-cell ALL (Figure 3D).

The subjects were divided into cohorts with high or low *CSRP2* transcript levels at the median *CSRP2* transcript value. All subjects with an *MLL* translocation and more subjects with a WBC > 30 × 10E+9/L were in the high *CSRP2* transcript level cohort (Table 2). There was no significant association between *CSRP2* transcript levels and age, sex, platelet level, percent bone marrow blasts, immune phenotype, *BCR-ABL1*, *IKZF1* deletion, risk group, MRD-test result at the end of induction therapy and/or post-remission therapy in multi-variate analyses (Table 2).

**Table 2:** Association of CSRP2 transcript levels with the clinical features of adult B-cell ALL

Variable	Total *N* = 168	L-*CSRP2 N* = 88	H-*CSRP2 N* = 80	*P*–value
Gender				0.895
Male	87	46	41	
Age (years)				0.275
Median	33	31	34	
Range	14–67	14–55	14–67	
Age (years) group				0.469
< 35	91	50	41	
WBC (×10E+9/L)				0.056
Median	10.6	8.6	15.5	
Range	0.3–586	0.3–523	1.3–586	
WBC group (×10E+9/L)				0.037
< 30	116	67	49	
Platelets (×10E+9/L)				0.483
Median	58.6	60.0	48.2	
Range	0.2–338	0.2–338	4.0–310	
Bone marrow blasts (%)				0.131
Median	88.0	86.5	89.0	
Range	20–99	20–99	22–97	
Immune phenotype				
Common-B-ALL	141	76	65	0.367
Pre-B-ALL	9	5	4	1.000
Pro-B-ALL	18	7	11	0.225
Cytogenetics				
Normal	56	34	22	0.126
*BCR-ABL1*	56	30	26	0.827
*MLL*-translocation	11	0	11	0.000
Hypo-diploidy	1	0	1	—
Complex cytogenetics	4	3	1	0.622
Other cytogenetics	40	21	18	0.834
*IKZF1*-deletion	77	37	40	0.301
Risk-group				0.141
High-risk	72	33	39	
Standard-risk	96	55	41	
MRD-test				0.234
Positive	97	47	50	
Chemotherapy-only	76	36	40	0.237

L-*CSRP2*, low transcripts.

H-*CSRP2*, high transcripts.

### *CSRP2* transcript levels, CIR and RFS

**Figure 4:**
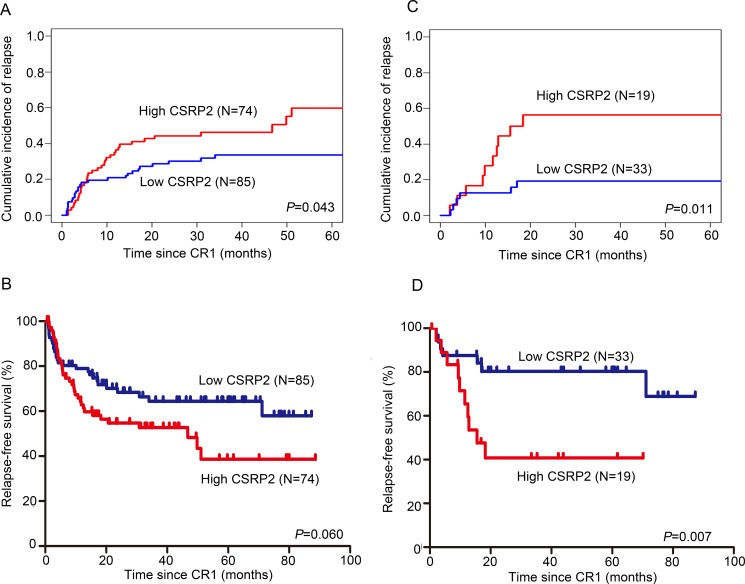
Association of *CSRP2* with CIR and RFS (**A**) Cumulative incidence of relapse (CIR); and (**B**) relapse-free survival (RFS) were compared between subjects with high or low *CSRP2* transcript levels. (**C**) CIR; (**D**) RFS were compared between subjects with normal cytogenetics with high or low *CSRP2* transcript levels.

Median follow-up was 20 months (range 1–90 months). Complete remission rates after one cycle of induction therapy in subjects with high and low *CSRP2* transcript levels were similar (84% [76, 92%] vs. 88% [81, 94%]; *P* = 0.488). Five-year CIR in subjects achieving remission with high *CSRP2* transcript levels was significantly higher than in subjects with low *CSRP2* transcript levels (60% [58, 61%] vs. 34% [33, 34%]; *P* = 0.043; Figure 4A). In subjects with low *CSRP2* transcript levels 5-year RFS was 64% (53, 76%) compared with 39% (22, 55%; *P* = 0.060) in subjects with high *CSRP2* transcript levels (Figure 4B). In multivariate analyses including age (≥ vs. < 35 years), gender, WBC (≥ vs. <30×10E+9/L), *BCR-ABL1* (N/Y), *MLL* translocation (no/yes) or IKZF1 deletion (N/Y), treatment (chemotherapy only vs. chemotherapy/allotransplant), MRD-test result at the end of induction therapy (negative/positive) and *CSRP2* transcript level (low/high), a negative MRD-test result, female sex, no *MLL* translocation and chemotherapy/allotransplant were associated with lower CIR (Table 3). Female sex, no *MLL* translocation and chemotherapy/allotransplant were associated with better RFS (Table 3).

**Table 3:** Multivariate analyses of CIR and RFS in adults with B-cell ALL

Outcome*^a^*	HR (95% CI)	*P*–value
**CIR**		
MRD Positive *vs*. Negative	1.16 (1.04–1.30)	0.010
Female *vs*. male	0.49 (0.28–0.86)	0.012
Transplant: Yes *vs*. No	0.28 (0.17–0.46)	0.000
*MLL* translocation: Yes *vs*. No	3.83 (1.64–8.91)	0.002
**RFS**		
Female *vs*. male	0.45 (0.26–0.76)	0.003
Transplant: Yes *vs*. No	0.24 (0.14–0.41)	0.000
*MLL* translocation: Yes *vs*. No	3.86 (1.61–9.28)	0.003

Abbreviations: HR, hazard ratio; CI, confidence interval.

^a^ Other variables with *P* > 0.1 (including High vs. low *CSRP2*) were sequentially excluded from the model.

### *CSRP2* transcript level is independently associated with CIR and RFS in subjects with normal cytogenetics

We next analyzed the prognostic impact of *CSRP2* mRNA expression in the 56 subjects with normal cytogenetics. Rates of complete remission after one cycle of induction therapy in subjects with high and low *CSRP2* transcript levels were similar (81% [64, 98%] vs. 83% [70, 95%]; *P* = 1.0). High *CSRP2* transcript levels were associated with a higher 5-year CIR (56% [53, 59%] vs. 19% [18, 20%]; *P* = 0.011; Figure 4C) and worse 5-year RFS (41% [17, 65%] vs. 80% [66–95%]; *P* = 0.007; Figure 4D) compared with subjects with low CSRP2 transcript levels. In multivariate analyses high *CSRP2* transcript levels were independently-associated with a greater CIR (HR = 5.32 [1.64–17.28]; *P* = 0.005) and worse RFS (HR = 5.56 [1.87, 16.53]; *P* = 0.002; Table 4). Female sex and chemotherapy/allotransplant were also associated with a lower CIR and better RFS (Table 4).

**Table 4:** Multivariate analyses of CIR and RFS in adult B-cell ALL with normal cytogenetics

Outcome*^a^*	HR (95%CI)	*P-value*
**CIR**		
High *vs*. low *CSRP2*	5.32 (1.64–17.28)	0.005
Female *vs*. male	0.13 (0.03–0. 67)	0.014
Transplant Yes *vs*. No	0.32 (0.14–0.74)	0.008
*IKZF1* deleted Yes *vs*. No	2.23 (0.96–5.16)	0.061
**RFS**		
High *vs*. low *CSRP2*	5.56 (1.87–16.53)	0.002
Female vs. male	0.14 (0.03–0.64)	0.011
Transplant Yes *vs*. No	0.33 (0.12–0.90)	0.030

Abbreviations: HR, hazard ratio; CI, confidence interval.

*^a^*Other variables with *P* > 0.1 were sequentially excluded from the model.

### *CSRP2* promotes cell proliferation *in vitro* and *in vivo*

**Figure 5:**
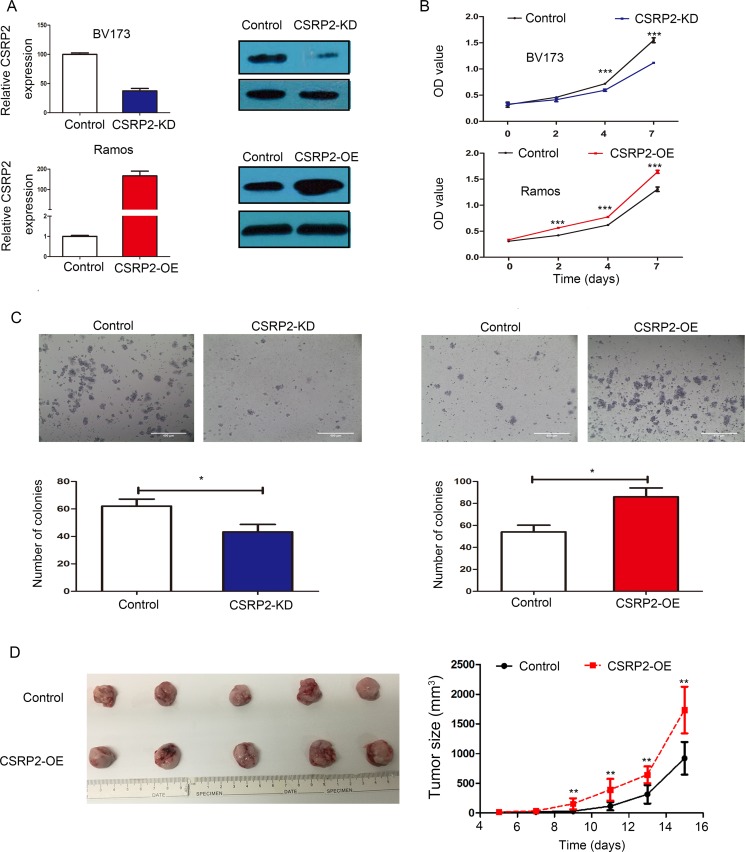
*CSRP2* promotes cell proliferation *in vitro* and *in vivo* (**A**) Knockdown efficiency of *CSRP2* in BV173 cells and ectopic expression of *CSRP2* in Ramos cells were demonstrated by RT-qPCR and western blot; (**B**) Knockdown of *CSRP2* significantly inhibited cell viability in BV173 and ectopic expression of *CSRP2* significantly enhanced cell viability in Ramos; (**C**) Numbers of colonies decreased when transfected with sh*CSRP2* in BV173 and ectopic expression of *CSRP2* increased numbers of colonies in Ramos; Size bar, 400 μm; (**D**) A representative picture of tumor formation in nude mice subcutaneously inoculated with *CSRP2*-OE Ramos cells or control Ramos cells (left panel); Tumor growth curves of *CSRP2*-OE Ramos cells and control Ramos cells in nude mice (Right panel). Values are mean ± standard deviation (SD). *P < 0.05; **P < 0.01; ***P < 0.001.

To study the biological role of *CSRP2* in B-cell ALL we developed 2 B-cell lines: (1) BV173 in which *CSRP2* was stably knocked-down (*CSRP2*-KD); and (2) a *CSRP2*-overexpressing Ramos cell line (*CSRP2*-OE; Figure 5A). Proliferation was significantly decreased in *CSRP2*-KD BV173 cells compared with cells transfected with control lentiviral particles. In contrast, *CSRP2*-over-expression in *CSRP2*-OE markedly- increased proliferation (Figure 5B).

We also tested whether *CSRP2* expression promoted colony formation. Silencing of *CSRP2* expression significantly decreased numbers of colony-forming units compared with controls. *CSRP2* over-expression had the converse effect (Figure 5C).

To further study the *in vivo* oncogenic activity of *CSRP2* unmodified Ramos cells and *CSRP2*-OE Ramos cells were injected subcutaneously into the dorsal right flank of nude mice. Tumors induced by *CSRP2*-OE Ramos cells were significantly larger than tumors induced by control Ramos cells (*P* < 0.01; Figure 5D). These data indicate *CSRP2* increases the oncogenicity of neoplastic B-cell lines *in vitro* and *in vivo*.

### *CSRP2* promotes cell-cycle progression and cell migration

**Figure 6:**
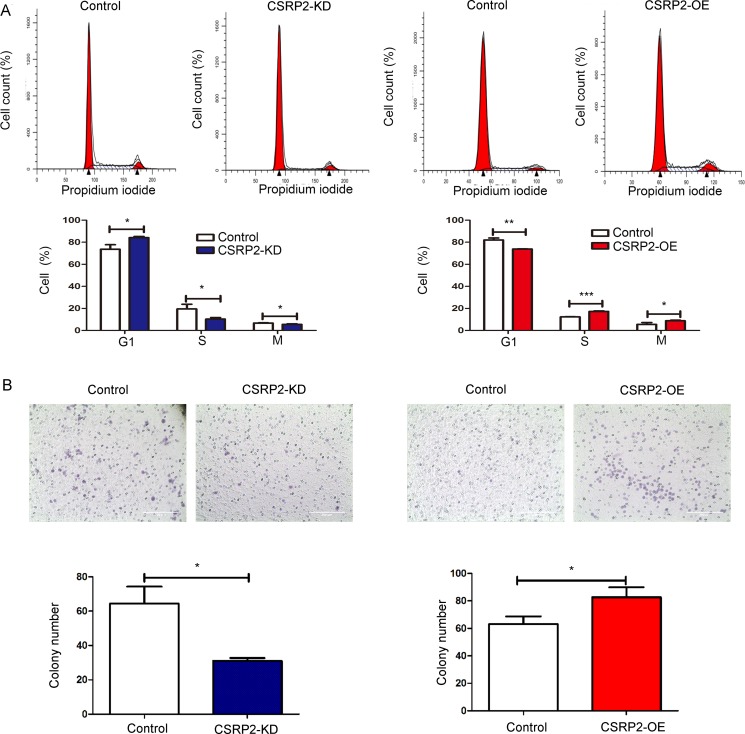
*CSRP2* promotes cell-cycle progression and cell migration (**A**) Cell cycle distribution was determined by flow cytometry; (**B**) Knockdown of *CSRP2* significantly inhibited cell migration of BV173 cells whereas ectopic expression of *CSRP2* significantly increased migration of Ramos cells. Size bar, 200 μm. Values are mean ± SD. *P < 0.05; **P < 0.01; ***P < 0.001.

Data from flow cytometry analyses showed increased *CSRP2* expression promote cell-cycle progression: *CSRP2*-OE cells in S and G2/M phases increased substantially compared with control cells whereas *CSRP2*-KD BV173 cells accumulated in G0/G1 phases compared with control cells (Figure 6A). Bio-informatic analyses showed *CSRP2* high expressing cells had increased cell-cycle progression (Table 5). *CSRP2* was moderately expressed in normal human B-cells (ARS = 43.16). In our previous study we reported moderately expressed genes had more plastic or variable expression in diverse experimental conditions [31]. The gene plastic (GPL) score of *CSRP2* was 19 in normal B-cells (400 GSMs) making it suitable for virtual sorting, an immune informatics method to evaluate immune cell subpopulations and their functions based on highly plastic genes.

**Table 5:** Functional annotation of *CSRP2* high expression B-cells

Term	Name	*P*-value	Fold-enrichment	Adjusted *P*-value (Bonferroni)
GO:0000279	M-phase	1.46E-07	7.32	1.21E-04
GO:0007067	Mitosis	2.35E-07	9.27	1.96E-04
GO:0000280	Nuclear division	2.35E-07	9.27	1.96E-04
GO:0022403	Cell-cycle phase	2.35E-07	6.27	1.96E-04
GO:0000087	M-phase of mitotic cell cycle	2.77E-07	9.10	2.31E-04
GO:0048285	Organelle fission	3.40E-07	8.90	2.84E-04
GO:0000278	Mitotic cell-cycle	5.14E-07	6.51	4.29E-04
GO:0007049	Cell-cycle	2.35E-06	4.06	1.96E-03
GO:0007059	Chromosome segregation	4.36E-06	16.01	3.63E-03
GO:0022402	Cell-cycle process	7.54E-06	4.59	6.27E-03
GO:0051726	Regulation of cell-cycle	6.18E-05	5.60	5.03E-02

The annotations are from biological processes of Gene Ontology (GO) *via* the DAVID website (https://david.ncifcrf.gov).

Migration activity of *CSRP2*-OE Ramos cells was greater than that of control Ramos cells. Knockdown *CSRP2* expression in BV173 cells eliminated their migration (Figure 6B).

### Subcellular localization of CSRP2 in neoplastic B-cells

**Figure 7:**
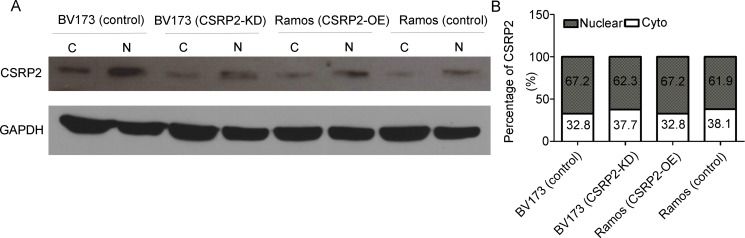
Subcellular localization of CSRP2 in neoplastic B-cells (**A**) Representative picture of immunoblots containing subcellular fractions; (**B**) Percent cytoplasmic and nuclear CSRP2 localizations were determined as follows: cytoplasmic CSRP2 density/(cytoplasmic CSRP2 density + nuclear CSRP2 density) × 100 and nuclear CSRP2 density/(cytoplasmic CSRP2 density + nuclear CSRP2 density) × 100, respectively.

We used cell fractionation analyses to assess subcellular localization of CSRP2 in neoplastic B-cells. CSRP2 protein was detected in the cytoplasm (C) and nucleus (N) and was more intense in the latter (Figure 7A). Silencing CSRP2 slightly decreased nuclear CSRP2 localization in BV173 cells whereas over-expression slightly increased nuclear localization in Ramos cells (Figure 7B).

### *CSRP2* knock-down increases drug-sensitivity

**Figure 8:**
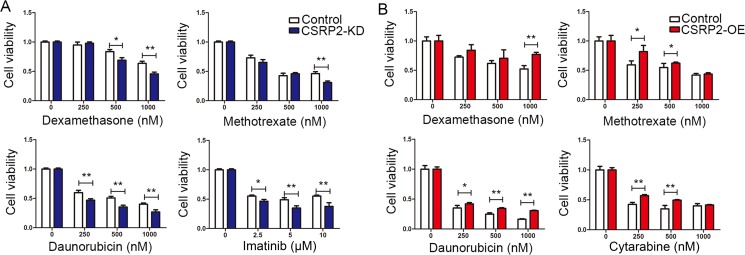
*CSRP2* knock-down increases drug sensitivity (**A**) Knockdown of *CSRP2* in BV173 cells significantly increased sensitivity to dexamethasone, methotrexate, daunorubicin and imatinib; (**B**) Ectopic expression of *CSRP2* in Ramos significantly increased resistance to dexamethasone, methotrexate, daunorubicin and cytarabine. Values are mean ± SD. *P < 0.05; **P < 0.01; ***P < 0.001.

Drug resistance is the main reason for treatment-failure and relapse in B-cell ALL. We studied the relationship between *CSRP2* transcript levels and B-cell ALL sensitivity to dexamethasone, methotrexate, daunorubicin, cytarabine and imatinib (in BV173 with BCR-ABL1). *CSRP2*-KD BV173 cells showed increased sensitivity to dexamethasone, methotrexate, daunorubicin and imatinib compared with controls (Figure 8A) whereas *CSRP2*-OE Ramos cells showed increased resistance to these drugs compared with controls (Figure 8B). These data suggest down-regulation of *CSRP2* may improve therapy-outcomes in adult B-cell ALL.

## DISCUSSION

We used bioinformatics-based analyses to identify potentially important genes transcribed in B-cell ALL. We focused on *CSRP2* because it was the most differentially expressed gene in our studies. *CSRP2* is a possible oncogene in hepatocellular carcinoma and breast cancer [17, 18]. *CSRP1* belongs to the *CSRP* family and is considered a tumor suppressor gene in hepatocellular carcinoma and colorectal cancer [32, 33] but a possible oncogene in gastric cancer [34]. In this study, we found *CSRP2* transcripts were uniformly low in bone marrow mononuclear cells from normals whereas transcript levels were high in cells from adults with newly-diagnosed B-cell ALL.

An *in silico* analyses using publicly available gene expression datasets reported worse survival of women with the basal-like subtype of breast cancer with high expression of *CSRP2* [18]. Our analyses of the prognostic impact of *CSRP2* transcript levels on CIR and RFS in adults with B-cell ALL was complex because of confounding with other prognostic variables and therapies. We used multivariate analyses to help resolve this complexity. CIR and RFS of subjects receiving transplants from different donors were similar so these data were combined. We found a positive MRD-test at the end of induction therapy, male sex, *MLL* translocation and chemotherapy-only were independently-associated with a higher CIR and worse RFS whereas other variables including age, WBC, *BCR-ABL1*, *IKZF1* deletion and *CSRP2* transcript levels were not. We discuss lack of a significant association between *BCR-ABL1* and CIR and RFS previously [35]. Although high *CSRP2* mRNA expression was associated with CIR in univariate analyses this association was not significant in multivariate analyses possibly reflecting confounding by high levels of *CSRP2* expression in persons with *MLL* translocation. Consequently, we re-analyzed an association between *CSRP2* transcript levels, CIR and RFS in subjects with normal cytogenetics. In multivariate regression analyses high levels of *CSRP2* transcripts were independently-associated with a higher CIR and worse RFS regardless of post-remission therapy.

In several experimental models of B-cell ALL increased *CSRP2* transcription promotes cell proliferation, migration and cell-cycle progression, a finding concordant with our bio-informatic analyses. Increased *CSRP2* transcription also promotes migration of breast cancer cells via an actin bundling factor [18]. In our study *CSRP2* was found predominantly in the nucleus. This contrasts with the cytoskeletal localization reported in breast cancer cells [18]. Moreover, knockdown of *CSRP2* transcription increased drug-sensitivity whereas increased *CSRP2* transcription increased drug resistance. These data suggest down-regulating CSRP2 transcription might decrease drug resistance and thereby decrease CIR and improve RFS. These conclusions are preliminary but warrant consideration.

There are several limitations to our study which was retrospective and susceptible to selection biases. The cohort with normal cytogenetics was not pre-specified and had relatively few subjects. Also, there is the potential for an interaction between *CSRP2* transcript levels and type of post-remission therapy. Because of these limitations our conclusion need validation in a larger, independent prospective cohort. If validated, determination of *CSRP2* transcript levels in adult B-cell ALL with normal cytogenetics might inform therapy-decisions. Also, consideration could be given to down-regulating *CSRP2* expression as a way to reverse drug resistance.

## MATERIALS AND METHODS

### Bioinformatics analyses

To identify possible B-cell ALL relevant genes data from the ImmuSort database dataset related to B-cell ALL and normal B-cell samples was updated and re-analyzed [9]. This database included gene expression profiles from >20,000 genes in human and mouse immune cells based on micro-array platform. Differences (delta values) in average rank score (ARS) were transformed from expressional signal value reflecting gene expression intensity. These data were used for gene expression comparison [9]. When a gene had multiple probe sets the probe with the maximum ARS was used.

### Cell lines

The human B-cell ALL cell lines BV173, Sup-B15 and BALL-1 were obtained from Guangzhou Jennio Biotech Co. Ltd (Guangzhou, China). The human Burkitt lymphoma cell line Ramos was a kind gift from Professor H.S. Zhao (Peking University Health Science Center, Beijing, China). The human T-cell ALL cell lines 6T-CEM and MOLT4, the human AML cell lines KG-1, NB4 and HL60, the human chronic myelogenous leukemia cell line K562 and the human lymphoma cell lines MAVER, U937, Raji and MOLP2 were available in our laboratory. Cell lines were cultured in Roswell Park Memorial Institute (RPMI) 1640 medium (Gibco, Billings, MT, USA) containing 10% fetal bovine serum (Gibco), penicillin (100 U/ml, Gibco) and streptomycin (100 μg/ml, Gibco). Cells were grown at 37°C in a humidified 5% CO2 atmosphere.

### Subjects

Bone marrow samples were obtained from adults with B-cell ALL (*N* = 236) and normals (*N* = 43) at the Hematology Department of Peking University People's Hospital, Beijing, China and *CSRP2* transcript levels assessed. Complete clinical and laboratory data were available for 168 subjects enrolled December, 2008 to June, 2014. Subjects were followed until death, loss to follow-up or June, 2016. The study was approved by the Ethics Committee of Peking University People's Hospital and informed consent was obtained according to the Declaration of Helsinki. Details of treatment regimens are reported [35]. 92 subjects (55%) received an allotransplant, 28 from an HLA-identical sibling, 1 from an HLA-matched-unrelated donor and 63 from HLA-haplotype-matched related donors [36, 37]. Complete remission, refractory disease, relapse and risk-stratification were defined as described [2]. Relapse-free survival (RFS) was determined from the date of first complete remission to the date of first relapse. Cumulative incidence of relapse (CIR) was determined from the date of first complete remission to the date of first relapse or death in complete remission.

### Immune phenotype, measureable residual disease (MRD) and cytogenetic analyses

Bone marrow samples were analyzed using standard four-color flow cytometry (FCM) [38]. Immune phenotypes were identified as: (1) early precursor B-cell ALL (pro-B-ALL) for CD10-negative, CD19-postive, cCD79a-postive, cCD22 positive and TdT-positive; (2) common B-cell ALL (common-B-ALL) for CD10-postive; and (3) precursor B-cell ALL (pre-B-ALL) for cytoplasmic μ+, sIg-, CD10+/− [2]. MRD was quantified by analyzing leukemia-associated aberrant immune phenotypes (LAIPs) using four-color flow cytometry as described [39]. A positive MRD-test was defined as ≥ 0.1% of cells with an LAIP phenotype in ≥ 1 bone marrow samples. Cytogenetic analyses were performed by G-banding [40]. *BCR-ABL1* transcripts and *MLL* rearrangements were detected with quantitative real-time polymerase chain reaction (RT-qPCR) [41, 42]. *IKZF1* deletions were detected as described [35].

### Lentiviral transduction

BV173 cells were infected with human *CSRP2* shRNA lentiviral particles (Santa Cruz Biotechnology, Santa Cruz, CA) or blank control lentiviral particles (Santa Cruz) at a 100 multiplicity of infection (MOI). Media containing lentiviral particles were replaced with complete medium 12 h post-infection and stably transfected BV173 cells were selected with 0.5 μg/ml puromycin dihydrochloride (Genechem, Shanghai, China) at 96 h post-infection. Ramos cells were infected with human *CSRP2* lentiviral activation particles (Santa Cruz) or control lentiviral activation particles (Santa Cruz) at a 100 MOI. Stably transfected Ramos cells were selected with 2.5 μg/ml puromycin dihydrochloride (Genechem), 15 μg/ml Blasticidin S HCl™ (Solarbio, Beijing, China) and 2000 μg/ml Hygromycin B™ (Solarbio). *CSRP2* expression levels were confirmed by RT-qPCR and western blot analyses.

### RNA preparation and RT-qPCR

Mononuclear cells were isolated from bone marrow samples by Ficoll-Hypaque™ density gradient centrifugation and RNA extracted using the TRIzol™ technique (Invitrogen, Carlsbad, CA, USA) according to the manufacturer's instructions and cDNA synthesized as described [43]. mRNA expression levels were analyzed using SYBR^®^ green (Applied Biosystems, Foster City, CA, USA) to validate differential expression screened out by bioinformatics analyses with *ABL1* or *GAPDH* as internal controls [44]. Gene transcript levels were determined using the 2^−ΔΔCt^ method. Average gene transcript levels in bone marrow samples from normals were used as calibrator. Other mRNA levels were determined by the TaqMan^®^ method [45]. *CSRP2* transcript levels were normalized to *ABL1* expression as recommended by the Europe Against Cancer group [46]. Copy numbers of *CSRP2* and *ABL1* were calculated from standard curves using the Ct values. Samples were assayed in duplicate to evaluate data reproducibility and average threshold Ct values calculated for expression analyses. Serial dilutions of plasmids expressing *ABL1* and *CSRP2*-positive bone marrow specimens were amplified to construct standard quantification curves [41]. These curves indicated similar amplification efficiency for *ABL1* and *CSRP2* with slopes of −3.50 and −3.49. Detection sensitivity was approximately 1–10 copies in the plasmid DNA standards and 10E-5 in *CSRP2*-positive bone marrow samples. For each measurement the curve threshold amplification was set at 0.08 for *ABL1* and *CSRP2*. Primers and probe sequences are shown in Supplementary Table 2.

### Western blot analyses

Western blotting was done as described [47]. Cytoplasmic and nuclear fractions were extracted as described [48]. The first antibodies were anti-*CSRP2* (rabbit monoclonal, 1:1000; Abcum, Cambridge, UK) and anti-GAPDH (rabbit monoclonal, 1:1000; Cell Signaling, Danvers, MA, USA) and the second antibody, horseradish peroxidase-conjugated goat anti-rabbit IgG (1:10000; Santa Cruz Biotechnology, Santa Cruz, CA, USA).

### Cell-cycle analyses

Cells were seeded to 6-well plates and starved by adding serum-free medium for G1 synchronization. After 24 hours, medium containing 10% fetal bovine serum was added for an additional 48 hours. Cells were fixed in 75% ethanol, stained with propidium iodide (BD Pharmingen, San Jose, CA, USA) and analyzed by flow cytometry. Results were analyzed with ModFit LT2.0 software (Coulter Electronics, Hialeah, FL, USA).

### Cell proliferation and viability assay

Cell proliferation was determined with the Cell Counting Kit-8 (CCK8, Dojin Laboratories, Kumamoto, Japan) assay. Briefly, 4 × 10E+4 cells were seeded into each well of 96-well plates. 2, 4 or 7 d later 10 μl of the kit reagent was added to each well and 2 h later all plates were scanned by a microplate reader at 450 nm. CCK8 was also used to determine cell viability after drug exposures including daunorubicin, dexamethasone, methotrexate, cytarabine and imatinib (Solarbio, Beijing, China). Cells were seeded and 72 h later 10 μl of the kit reagent was added to each well and 2 h later plates were scanned by a microplate reader at 450 nm. Cell viability was assessed based on the value of fluorescent signal of live cells with no drug treatment. Experiments were performed in triplicate for 3 times independently.

### Colony formation assays

Cells were suspended in 1 mL of complete MethoCult™ medium and plated in 6 well plates at a concentration of 4 × 10E+3 /well. Colonies were maintained at 37°C with 5% CO2 and 95% humidity for 7 d and then counted and scored at day 7 after staining with 1% crystal violet (Sigma, St. Louis, MO, USA). Only colonies of ≥ 50 cells were scored. Assays were done in triplicate for 3 times independently.

### Cell migration assay

Cells were seeded into the upper chamber of a Transwell insert (pore size, 8 μm) in RPMI-1640 supplemented with 1% FBS. The upper chamber was then placed into the Transwell containing medium with 10% FBS in the lower chamber. After 24 h, cells remaining in the lower surface of the insert were stained with crystal violet. Experiments were conducted in triplicate for 3 times independently.

### Tumor xenograft mouse model

Male athymic 6-week-old Balb/c nude mice (Beijing HFK Bioscience Co., Ltd.; Beijing, China) were housed in a controlled environment with a 12 h light/dark cycle at 23°C (± 2°C) and 40–50% relative humidity with free access to chow and water. Animal experiments were approved by the Animal Ethics Committee of Peking University Health Science Center. Mice were pretreated by intraperitoneal injections of cyclophosphamide once daily at a dose of 100 mg/kg for 2 consecutive days. Two days later, Ramos cells (1.5×10E+7 cells in 0.1 mL PBS) transduced with a lentivirus containing *CSRP2* lentiviral activation particles or control lentiviral activation particles were injected subcutaneously into the dorsal right flank of 6-week-old male Balb/c nude mice (5 mice/group). Tumor diameters were measured every 2 days until day15. Tumor volume (mmE+3) was estimated by measuring the longest and shortest diameter of the tumor as described [49]. Mice were euthanized on day 15 and tumors surgically removed and photographed.

### Statistical analyses

Differences across groups were compared using the Pearson Chi-square analysis or Fisher exact test for categorical data and Mann-Whitney *U* test or Student *t-test* for continuous variables. Receiver operating characteristic (ROC) curves were constructed to evaluate the predictive power of transcript levels for diagnosis of B-cell ALL. The Youden Index was used to calculate optimal cutoff points for gene transcript levels in diagnosis of B-cell ALL [50]. Survival functions were estimated by the Kaplan-Meier method and compared by the log-rank test. Cumulative incidences were estimated for relapse to accommodate competing risks. A Cox proportional hazard regression model was used to determine associations between *CSRP2* transcript levels and CIR and RFS. Variables with *P* > 0.1 were sequentially excluded from the model and those with a *P* < 0.05 considered significant. A two-sided *P* < 0.05 was considered significant. Analyses were performed by SPSS software version 18.0 (Chicago, IL, USA), Graphpad Prism™ 5.01 (San Diego, California, USA), OriginPro 9.2 (Wellesley Hills, MA, USA), SAS 9.4 software (SAS, Cary, NC, USA) and R software package (version 3.1.2;http://www.r-project.org).

## SUPPLEMENTARY MATERIALS FIGURES AND TABLES



## Abbreviations

ALL: Acute lymphoblastic leukemia
*CSRP2*: Cysteine and glycine-rich protein 2
RT-qPCR: quantitative real-time polymerase chain reaction
CIR: cumulative incidence of relapse
RFS: relapse-free survival
MRD: measureable residual disease
ARS: average rank score
RPMI: Roswell Park Memorial Institute
FCM: flow cytometry
pro-B-ALL: early precursor B-cell ALL
pre-B-ALL: precursor B-cell ALL
LAIPs: leukemia-associated aberrant immune phenotypes
MOI: multiplicity of infection
CCK8: Cell Counting Kit-8
GEO: Gene Expression Omnibus (GEO)
ROC: Receiver-operator characteristic
GPL: gene plastic
