# Supplementary material for: Cysteine and glycine-rich protein 2 (CSRP2) transcript levels correlate with leukemia relapse and leukemia-free survival in adults with B-cell acute lymphoblastic leukemia and normal cytogenetics
Source: Oncotarget. 2017 Mar 21;8(22):35984–6000. doi: 10.18632/oncotarget.16416 (PMC5482632; doi:10.18632/oncotarget.16416)
Supplement: Supplementary file 1 [file oncotarget-08-35984-s001.pdf]

Cysteine and glycine-rich protein 2 (*CSRP2*) transcript levels correlate with leukemia relapse and leukemia-free survival in adults with B-cell acute lymphoblastic leukemia and normal cytogenetics

Supplementary Materials

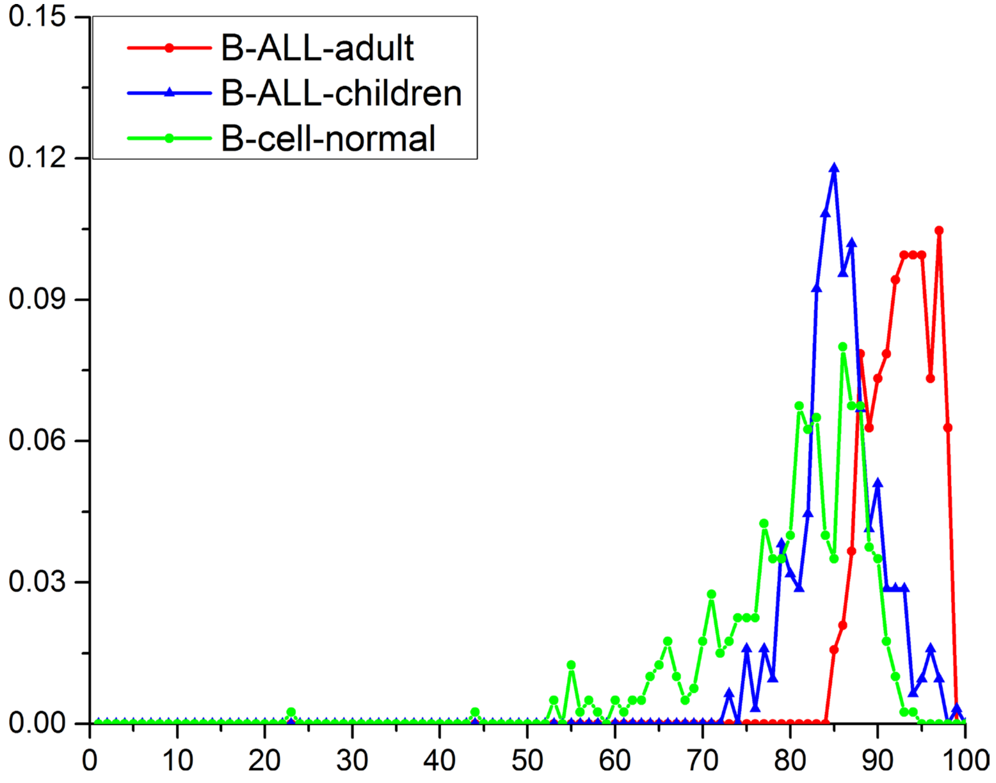

Supplementary Figure 1: Gene expression profiles of the *ABL1* gene in adult and childhood B-cell ALL. The rank-based gene expression (RBE) curves in the ImmuSort database indicate sample distribution in term of gene expression across various individuals and experimental conditions. The x-axis represents the percentile rank scores from 1 to 100 with increasing expression intensity. The y-axis represents the sample proportion at an indicated rank score. Right and left peaks indicate high and low expression.

Supplementary Table 1: Expression levels of *CSRP2* in leukemia and lymphoma cell lines

Cell line	MIC[a]	Number of repetitions	*CSRP2/ABL1*%[b]
BV173	B-cell ALL	3	397.89 ± 46.57
Sup-B15	B-cell ALL	3	512.94 ± 71.46
BALL-1	B-cell ALL	3	43.67 ± 5.03
6T-CEM	T-cell ALL	3	0.17 ± 0.01
MOLT4	T-cell ALL	3	1.11 ± 0.06
NB4	Promyelocytic leukemia	3	0.02 ± 0.01
HL60	Promyelocytic leukemia	3	0.32 ± 0.17
KG-1	AML	3	3.05 ± 0.43
K562	CML, blast crisis	3	0.06 ± 0.00
MAVER	Mantle cell lymphoma	3	309.84 ± 31.57
U937	Histiocytic lymphoma	3	1.13 ± 0.50
Raji	Burkitt lymphoma	3	0.005 ± 0.002
Ramos	Burkitt lymphoma	3	1.56 ± 0.23

[a]MIC: morphology, immunology and cytogenetics classification.
[b]Data is presented as mean ± SD.

Supplementary Table 2: Sequences of primers and probe used in this study

Name	Sequence (5′–3′)
CSRP2-Forward primer	GTGATGGCAGGAGCTTCCA
CSRP2-Reverse primer	GCCACTGTTGTGCTATCTAAATTTTT
CSRP2-Probe	FAM-CGCTGCTGCTTTCTCTGCATGGTTT-BHQ
ABL1-Forward primer	CCGCTGACCATCAATAAGGAA
ABL1-Reverse primer	GATGTAGTTGCTTGGGACCCA
*ABL*1-Probe	FAM-CCATTTTTGGTTTGGGCTTCACACCATT-TAMARA
GNA15-Forward primer	CTACCAGAACATCTTCGTGTCCAT
GNA15-Reverse primer	GCTGAATCGAGCAGGTGGAA
C19orf77-Forward primer	CACCCCTCTTCCTGAGCTTG
C19orf77-Reverse primer	GATTGCGGGTGTCTCCTCAA
COL5A1-Forward primer	CCAAAGAAAACCCGGGCTCC
COL5A1-Reverse primer	GCAGTGGTAGGTGACGTTCT
FRMD4B-Forward primer	TCGAAGTAGAGAGCGAAACCATC
FRMD4B-Reverse primer	CTTGACCTCGAGTGAGACCTTTG
CPNE2-Forward primer	CATTCACAGTGCCCTTGGTGTCCCTGT
CPNE2-Reverse primer	GTCTCGGTTTATCTTGCAGGATC
HBEGF-Forward primer	GTATCCACGGACCAGCTGCTA
HBEGF-Reverse primer	CCTTGTATTTCCGAAGACATGG
C5orf62-Forward primer	GGATGCAGTCAGCCAAGTCC
C5orf62-Reverse primer	AGCCCCACTAAGGATCGGAT
RASD1-Forward primer	TCATCCTGGTGTTCAGTCTGG
RASD1-Reverse primer	AGGCAAGACTTGGTGTCGAG
GAPDH- Forward primer	AGAAGGCTGGGGCTCATTTG
GAPDH- Reverse primer	AGGGGCCATCCACAGTCTTC
